# V4RIN: visual analysis of regional industry network with domain knowledge

**DOI:** 10.1186/s42492-024-00164-9

**Published:** 2024-05-15

**Authors:** Wenli Xiong, Chenjie Yu, Chen Shi, Yaxuan Zheng, Xiping Wang, Yanpeng Hu, Hong Yin, Chenhui Li, Changbo Wang

**Affiliations:** 1 School of Computer Science and Technology, East China Normal University, Shanghai, 200062 China; 2 China Fortune Securities Co., Ltd, Shanghai, 200030 China; 3 Shanghai Chinafortune Co., Ltd, Shanghai, 200030 China; 4 Faculty of Economics and Management, East China Normal University, Shanghai, 200062 China

**Keywords:** Visualization, Visual analytics, Visualization in finance, Regional industry analysis

## Abstract

The regional industry network (RIN) is a type of financial network derived from industry networks that possess the capability to describe the connections between specific industries within a particular region. For most investors and financial analysts lacking extensive experience, the decision-support information provided by industry networks may be too vague. Conversely, RINs express more detailed and specific industry connections both within and outside the region. As RIN analysis is domain-specific and current financial network analysis tools are designed for generalized analytical tasks and cannot be directly applied to RINs, new visual analysis approaches are needed to enhance information exploration efficiency. In this study, we collaborated with domain experts and proposed V4RIN, an interactive visualization analysis system that integrates predefined domain knowledge and data processing methods to support users in uploading custom data. Through multiple views in the system panel, users can comprehensively explore the structure, geographical distribution, and spatiotemporal variations of the RIN. Two case studies were conducted and a set of expert interviews with five domain experts to validate the usability and reliability of our system.

## Introduction

Industry network refers to the network structure formed by different industries through connections such as supply chains and technology transfers. However, the wide range of entities within an industry network makes it challenging to provide detailed information at the geographical level. Consequently, regional economic specialists are increasingly analyzing the regional industry, which includes firms within a specific economic region that produce similar products or engage in the same type of industry.

A regional industry network (RIN) comprises the regional industries linked through economic interdependencies. Such networks offer in-depth insights for investors and analysts, uncover concerns not evident in broader industry analyses, and aid in understanding industry interactions within a region. Current research on RINs is primarily concentrated in the field of economics, where researchers generally use statistical methods like factor analysis to study the economic elements involved. Additionally, visualization researchers have designed visual analysis tools specifically for analyzing regional industry entities. For example, to analyze individual regional industries, Chen et al. [1] introduced RISeer, which combines the visual representations of bubble-like graphs and connecting lines to help users visually analyze the evolutionary dynamics of the structure of a RIN. To analyze the industrial connections within a region, Hermes conducted a visual analysis of the cash flow network, revealing the economic connections between productive sectors and geographic regions [2]. Sabrina employed multi-scale visual analysis views to help experts discern potential business associations among industrial entities [3]. However, most of these studies tended to emphasize the regional attributes attached to industries and then analyze the industrial structure within individual regions or the distribution status of industries across regions; that is, they focused more on the regional characteristics of industries rather than analyzing the economic relationships between regional industries as distinct entities. In summary, the analysis of the relationships between regional industries is an important research topic in the analysis of the entire RIN, and existing related works lack the ability to explore the RIN from a more diversified perspective.

To address this research gap, we collaborated with several domain experts. After fully collecting the analysis demands of RINs in reality, we decided to design a novel domain knowledge-oriented visualization solution for some specific RIN analysis tasks, which would help researchers interactively explore regional industry entities as well as associative relationships in coordinated visualization views. After understanding the specific domain tasks, the design process of the proposed solution faced several challenges. First, highlighting the geographical and industrial characteristics of regional entities is necessitated for solving all analytical tasks. However, designing visual analysis methods that can mine potential patterns from both the geographic distribution and network structures is challenging. Effective visual representations must help experts to clearly identify potential patterns in data across spaces without creating logical complexity or visual confusion. Second, to meet the practical analytical needs that experts face, comprehensive visualization panels generated from multi-dimensional, multi-source data, often contain excessive amounts of information, which can lead to visual clutter and complex operation. Efficient visual design should not only rationally filter and refine data to prevent information overload but also achieve a balance between the details of the views and the intuitiveness of the layout, enabling experts to focus on reliable and critical insights.

For the first challenge, we proposed innovative visualization designs based on conventional graph-based geovisualization methods, integrating techniques such as multi-layer switching and convex hull algorithms. These support experts in exploring distribution and connection patterns of regional industrial entities within both geographical and network spaces simultaneously and reveal the impact of entity characteristics on RINs. To address the second challenge, we developed a multi-perspective collaborative visual analytic system that designs views with distinct functions for different analytical tasks. The layout and interactive operation of the views are user-friendly, making it easy for financial experts to perform their tasks. Case studies and expert reviews of real-world scenarios demonstrated the usefulness of the proposed system. We believe that this is a new visual analytic study designed for specific RIN analysis tasks. In summary, this study makes the following contributions:

- We summarized a series of main analysis tasks of RINs from qualitative interviews with domain experts and designed diverse visual solutions accordingly.
- We have developed a multi-view collaborative visual analytics tool that supports comprehensive exploration of entities and relationships within RINs.
- We validated the interpretability and applicability of our proposed visualizations by conducting two case studies and interviews with six experts.

### Visual analysis of financial network

Numerous entities and relationships, such as transaction behavior, asset relationships, and market fluctuations, are involved in the financial domain. A financial network is a structure used to represent complex network relationships among these financial entities, typically composed of financial entities as nodes and the financial relationships between entities as edges. Researchers can obtain new insights from analyzing financial networks to make informed decisions [4, 5]. Visual analytics is a method of presenting data in graphical or other visual formats so that people can understand and analyze the data more easily. Compared to other data analysis methods, the visual analysis of financial data can further help people understand these complex financial relationships and discover potential patterns to support tasks such as financial decision-making, risk management, and market monitoring.

In recent years, visual analysis approaches for financial networks have been broadly classified into two categories from the perspective of task objectives. The first category identifies potential patterns or assesses the contagion risk in a network based on predefined rules. Leite et al. [6] developed a visual analytic system that supports network exploration and proposed the solution for pattern analysis and fraud detection on bank transaction network data. Niu et al. [7] assessed the risk of nodes in interbank transaction networks and developed a visual analysis system that enables users to assess, intervene, and mitigate risks in financial networks. Some researchers have combined pattern analysis and fraud detection. For example, Niu et al. [8] proposed visual analytic solutions for default contagion risk in interbank guarantee networks, thereby facilitating the discovery of contagion chain patterns.

The second category explores the node attributes and relationships of the financial network. Arleo et al. [3] visualized the variability of indicators and the spatio-temporal evolution of the relationships between objects. Similarly, Chen et al. [1] proposed an interactive visualization system with new visual features to analyze the evolution process of the regional industry structure. Both these studies provided insights into financial networks from the perspective of geographical distribution. They utilized geovisualization methods to present financial networks on maps, enhancing researchers’ understanding of the geographic attributes of the nodes as well as the distribution of relationships in the financial network. In particular, RISeer, proposed by Chen et al. [1], is one of the few visual analysis works designed for regional industries. It revealed the current state of the regional industry structures and their dynamic changes over time from a microscopic perspective. Inspired by this study, we designed a visual analysis tool for RINs primarily from a geographical perspective to emphasize the most important regional distributional characteristics in the analysis of RINs.

While there are several visual analyses of financial networks designed for different research purposes, few deal with regional industries as financial entities; they focus more on designing applicable visualizations to show the geographical distribution of industries across regions. These approaches ignore the important economic linkages that exist between regional industries, which are a class of financial entities. Analyzing the structure of RINs can reveal key economic linkages and competition within and outside the region. Therefore, designing visual analytical tools for this purpose has practical significance and application value.

### Graph-based geovisualization

Because financial data containing geographic attributes are typically complex, heterogeneous, and large-scale, the expression of geovisualization must be augmented by leveraging the graph structure.

Graph-based geovisualization is a research field that concentrates on the visualization of geographic networks through graph-based methodologies [9, 10]. Chen et al. [11] drew the road network on the map, supporting users to query spatial-temporal and social interconnectedness features of real urban data. Similarly, Huang et al. [12] encoded spatio-temporal information from a vast amount of trajectory data into a 2D map-based network and simplified the exploration and querying processes of mobile phone user trajectories.

Nevertheless, our work is concerned with the evolution of the relationships between entities rather than the description of the spatial and temporal evolution of individual entities, such as the movement of entities in a trajectory network. Such work [13–16] is commonly found in the analyses of urban and social media data. However, few studies have been conducted on financial network data with geographic information. Wang et al. [17] adopted several methods to construct a regional map based on geo-textual data and designed different visual views to show regional migration trajectories. Arleo et al. [18] utilized a hexagonal design to visually represent individual companies on a map and displayed the financial transaction relationships between them in 3D space.

As more network data with geographic information are studied, the proposed visual approach must minimize the visual conflict between network elements and geographical distribution while highlighting distinct visual presentation emphases. An interactive visualization analysis system for regional industry network (V4RIN) is a new geovisualization tool for RINs, offering a series of efficient and effective collaborative visual analytics solutions.

## Methods

### Requirement analysis

As a cross-disciplinary issue, the demand for research on RINs originates from financial analysis, and the corresponding solutions are drawn from data analysis and visualization. We collaborated with nine experts, with E4-E9 primarily involved in collecting user experience feedback. Further details will be presented in Expert review subsection. E1 and E2 are investment research analysts with six and eight years of experience in financial institutions, respectively, whereas E3 is a PhD with over five years of experience in financial data visualization and graph learning. From August 2022 to March 2023, we held weekly discussions lasting approximately one hour with experts E1 and E2, while maintaining a more frequent face-to-face views exchange with expert E3. The discussions focused on the difficulties that experts may have encountered in analyzing RINs as well as questioning and evaluating the current process of system design and implementation.

After continuous in-depth communication, experts highlighted the application value of a system that integrates multiple visual analysis approaches to help researchers understand complex data. E1 expressed that, as a solution for financial analysis tasks, it is crucial to maintain the integrity and accuracy of the data. This implies that the designed visualization methods should avoid distorting data, such as introducing arbitrary precision loss in numerical values or using color schemes that may misrepresent differences in data. E2, with experience in designing financial systems, suggested that while the analysis of RINs should primarily focus on the relationships between each regional industry and its geographical distribution, exploring deeper into the economic attributes of regional industries may provide users with more insightful perspectives. For instance, he would be more concerned about the service industry in Shanghai being closely associated with industries in Jiangsu Province or Zhejiang Province, which affects his investment choices. Conversely, E1 mentioned that, based on his analytical experience, being able to observe the geographical distribution changes of various regional industry entities without frequently switching views is crucial for understanding market trends. Furthermore, when presenting other financial visual analytic systems to experts, they noted that, although these systems lacked depth in terms of interaction and included information, effective visual analysis tools remain an outstanding choice to ease the burden on researchers. E3 pointed out that they were often overwhelmed by various complex operations when using other industry analysis systems in the market. Some systems provide excessively detailed information, requiring users to spend a significant amount of time filtering and understanding the data. E1 added that most systems only present the final results to experts and that data quality is not easy to verify. Having a module in our system that provides explanatory results would be more beneficial for analysts to understand the RIN. In summary, experts require visual analytics methods that integrate map presentation, spatio-temporal observation, categorical display, drill-down into indicators, and other functions to accomplish key domain tasks accurately and efficiently. Specifically, we summarize the main requirements in the following five points:

- **R1: Present the RIN intuitively.** The experts focus on regional industries that exhibit close relationships, which typically imply significant interrelationships between these regional industries. The structure of RINs should be presented to experts in an intuitive and comprehensible manner, such as using connecting lines to represent economic relationships directly to facilitate experts’ better understanding of these relationships and enable them to quickly compare regional industries in which areas have relatively close economic relationships.
- **R2: Explore regional industry characteristics in a manner that aggregates important information.** Extensive data are involved in the analysis of RINs, such as various economic indicators for regional industries, the weights of relationships between regional industries, and records of changes in the geographical coordinates of regional industries. Noise inevitably exists and may interfere with expert judgment. Experts desire visualization methods that can centralize and highlight crucial information to help alleviate potential visual clutter and interaction difficulties.
- **R3: Analyze high-dimensional semantic similarity of nodes in RINs.** As economic entities, nodes in RINs often possess unique economic characteristics. A thorough understanding of these economic characteristics is crucial for interpreting and analyzing regional industries. However, the specific meanings of these features often lie in high-dimensional spaces, making it difficult to observe their distributions directly. Therefore, it is necessary to transform the high-dimensional semantics of node features into a low-dimensional space and to support experts in exploring their similarities in the low-dimensional space.
- **R4: Explain the temporal and spatial dynamics of RINs.** The temporal and spatial dynamics of RIN can reveal the rise and fall of regional industries over time and space. For example, with the introduction of policies and regulations, specific regions may attract more enterprises to specific industries. Therefore, further investigation is needed to discover the general patterns and states of change in RINs using dynamic temporal and spatial data.
- **R5: Analyze the regional industry entities and their relationships from multiple perspectives.** Because the node entity of the RIN is the regional industry that inherently possesses regional and industrial characteristics, experts believe that the analysis of RIN requires integrating both regional and industry perspectives. Thus, it is necessary to design coordinated interactions that combine multiple analysis views to enhance the richness of the presented information.

### Models

**Fig. 1:**
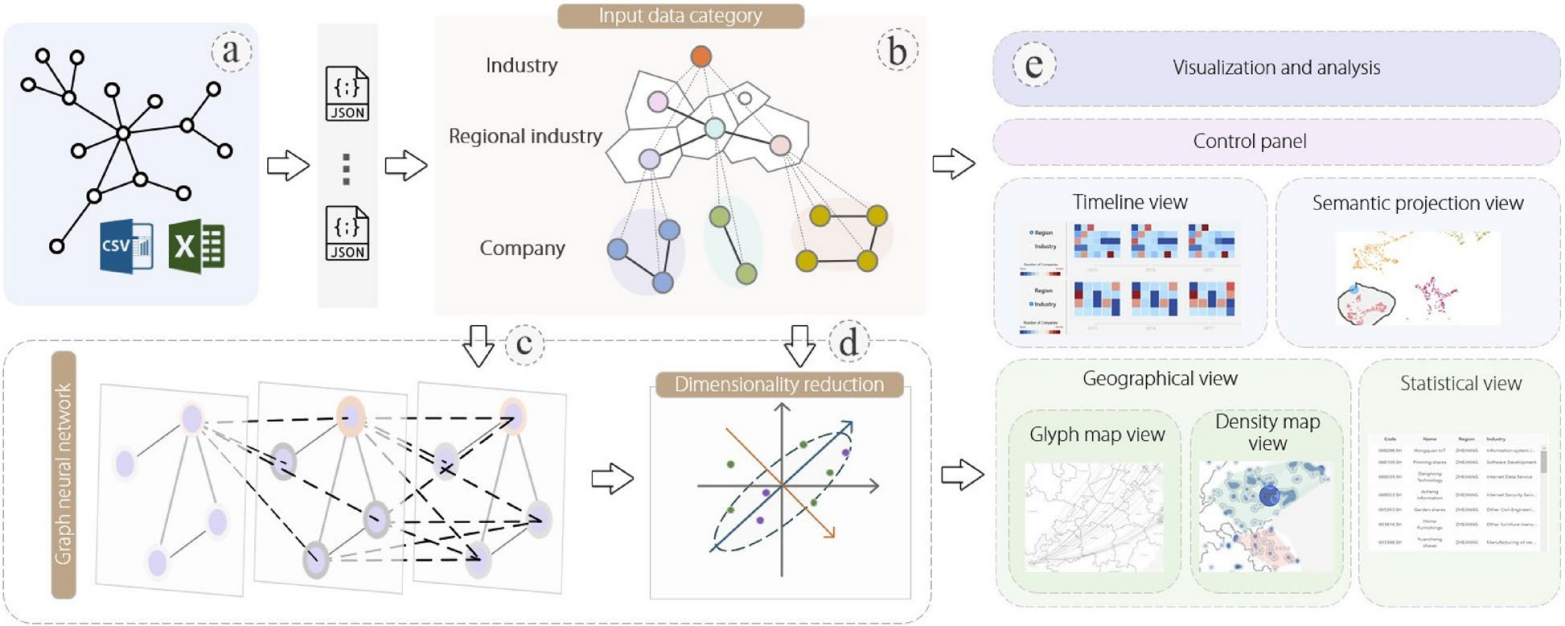
The pipeline takes structured and semi-structured data (**a**) used to represent the RIN (**b**) and parses it into JavaScript Object Notation (JSON) format. The backend integrates graph neural network (GNN) methods (**c**) to encode node information in the network. Then, the encoded vectors are mapped to a low-dimensional space through dimensionality reduction methods (**d**). The visual analytic system (**e**) is developed with the guidance of experts

The proposed visualization solution is for RIN data. Inspired by works such as RISeer, the proposed solution integrated the data processing and analysis model into the backend of the visual system to achieve end-to-end data presentation. The proposed pipeline, as illustrated in Fig. 1, is divided into two modules: backend data processing and frontend visual presentation.

The system initially parses the input data that characterize RINs in JSON format. This type of data (Fig. 1a) primarily consists of structured data that can be processed using relational databases, including network structures and regional industry economic indicators. Users can also independently process other semi-structured data; subsequently, by adding the processed features to the input data, our system automatically recognizes them as features of nodes or edges in the network and incorporates them into the next processing step. During the data parsing process, a series of basic data cleaning approaches were conducted, such as merging redundant information, removing outliers, and interpolating to supplement missing values. To enhance the applicability of the system, excessive feature filtering was avoided, and maintaining the integrity of the input data was strived for.

The processed JSON file represents the RIN, as shown in Fig. 1b. As previously mentioned, RINs are financial networks with geographical attributes. For the global visual representation (R1) of such networks, we prefer a graph-based geovisualization approach. The proposed system provides a panel called a geographical view that maps the network nodes onto a map based on the input geographical attribute data. Relationships are visualized on a map in the form of solid lines. Additionally, convex hulls based on the upper-level industry labels of the nodes were calculated, allowing users to observe the data distribution from a global perspective. Semantic visualization [19] combined with knowledge graph mining methods [20] refers to the extraction of visual relationships between knowledge from big data, which helps enhance hidden knowledge discovery and reasoning [21]. Therefore, in addition to globally observing geographical layouts, the GNN method (Fig. 1c) was utilized to encode the RIN and extract crucial semantic information from it. Each regional industry node *v*_*i*_ is encoded as a 16-dimensional vector ${h}_{{v}_{i}}$. The information propagation mechanism of GNN methods ensures that ${h}_{{v}_{i}}$ contains the features of the node in the graph structure, information from neighboring nodes, and global information of the graph. Therefore, ${h}_{{v}_{i}}$ is used as a vector that characterizes the semantics of the node *v*_*i*_. Semantics in this paper refer to the key features of nodes in the RIN, and the specific meaning depends on the input network properties and the application domain. Because experts expressed the demand for an interactive approach that highlights key information in Requirement analysis subsection, ${h}_{{v}_{i}}$ was mapped onto a two-dimensional space through dimensionality reduction (Fig. 1d). Specifically, two-dimensionality reduction methods, Umap and t-SNE, were internally integrated, and they provided a panel called the semantic projection view to visualize the distribution of nodes after dimensionality reduction; this helps users quickly grasp intuitive impressions of the semantic relationships among regional industry nodes, thereby enhancing the interpretability of the original data patterns. Finally, our visual solution is implemented as a visual analytic system (Fig. 1e), allowing users to explore data flexibly and comprehensively through interactive operations. The design and functionality of the system are introduced in System design and implement subsection.

### System design and implement

**Fig. 2:**
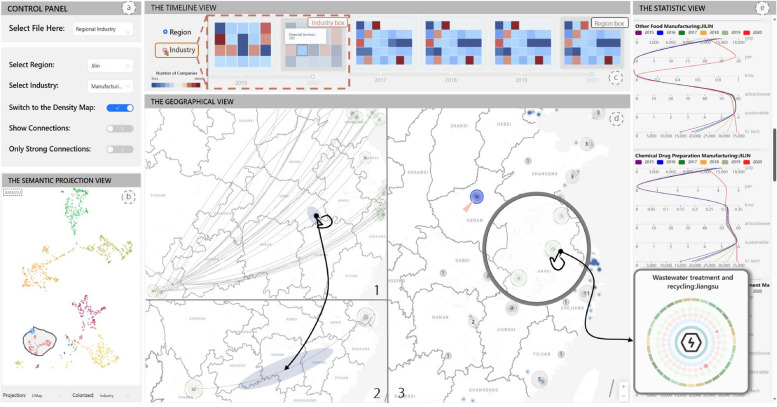
The system interface of V4RIN: **a** Control panel, providing data uploading and filtering functions; **b** Semantic projection view, visualizing the semantics of regional industries in two dimensions; **c** Timeline view, providing a selection of time points and visualizing trends in the number of companies in each region and industry over time; **d** Geographical view, visualizing the geographical distribution of regional industries and their interrelationships in the form of maps; **e** Statistical view, presenting statistical information about economic entities from multiple perspectives

We developed a web application based on the characteristics of RINs and the above framework (R5). As shown in Fig. 2, the entire system mainly consists of four parts: (1) The semantic projection view provides a visualization of the two-dimensional distribution of semantic vectors, where each node represents a specific regional industry. With a lasso operation, users can select multiple nodes simultaneously, whereas other views are linked to present detailed information after the selection. (2) The geographical map view depicts the geographical distribution of regional industries and the relationships among them on the map from two aspects. The glyph map view shows novel metaphorical symbols that represent the aggregation of information regarding relationships. The density map view presents the geographical distribution of each regional industry in the form of a heatmap marked by the maximum area mapping algorithm. (3) The timeline view provides an enhanced timeline where the color shades of each box encode the number of companies in a region or industry at the current time. (4) The statistical view presents some basic information about regional industries via parallel coordinates and data tables.

#### Semantic projection view

The Semantic projection view subsection provides a two-dimensional scatterplot to enhance the ability of experts to analyze and understand high-dimensional data. As mentioned in Requirement analysis subsection, exploring the semantics’ distribution of each regional industry is important, but difficult to conduct (R3). We reduced the high-dimensional semantic features to a two-dimensional space. Based on the dimensionality reduction results, we designed a semantic projection view to satisfy the experts’ requirement for interpretability.

**Fig. 3:**
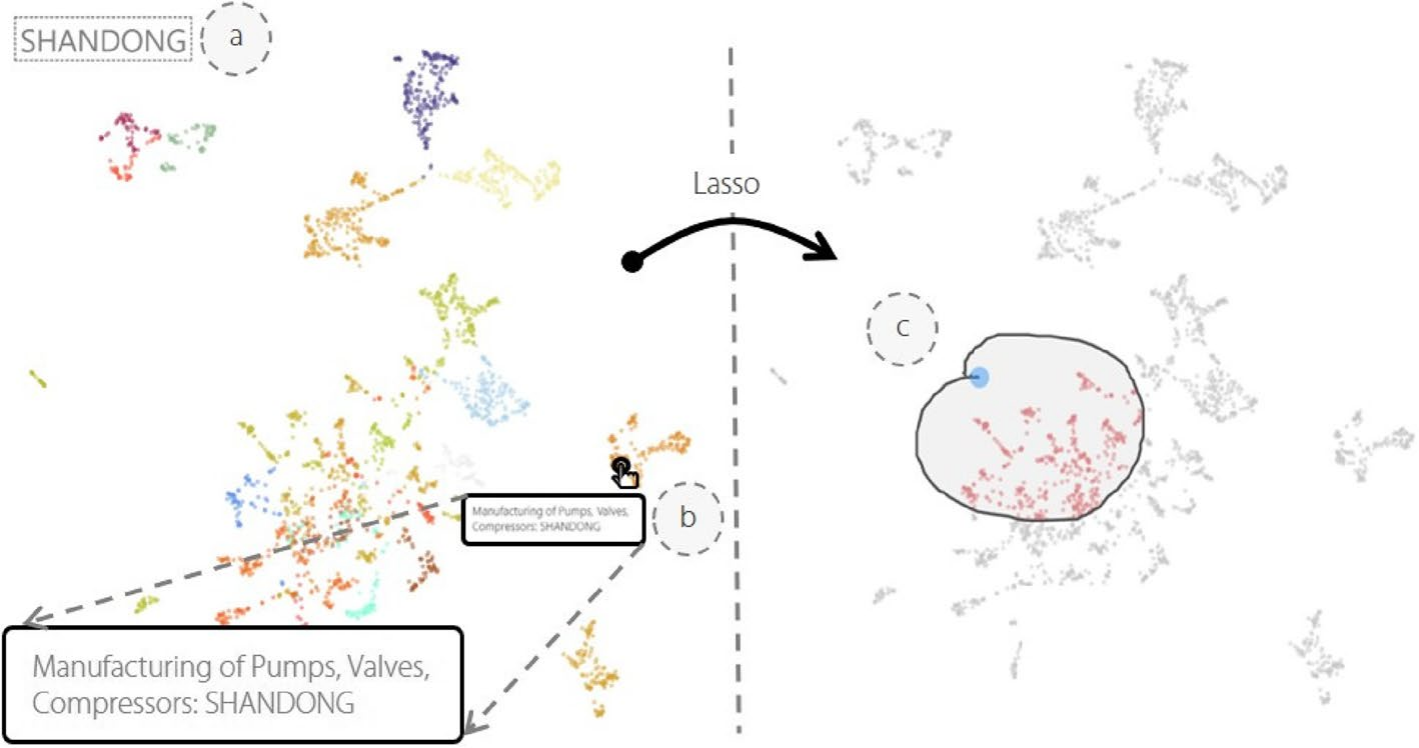
The semantic projection view provides several interactions, such as mouse hover (**a**), mouse click (**b**), and lasso operation (**c**)

Several dimensionality reduction methods are available such as Umap [22], t-SNE [23], LDA [24], and LLE [25]. Umap and t-SNE outperformed the other methods in terms of processing speed, number of adjustable parameters, and preservation of local structure information. Therefore, we utilized Umap and t-SNE to transform high-dimensional semantic vectors into a two-dimensional space. The reduced dimensional representation is displayed in a scatterplot, as illustrated in Fig. 3, where each point represents a specific regional industry, and the color of each point indicates its label (industry or region, depending on the user’s selection in the switching selector). Points that are spatially closer together have relatively closer semantics extracted from high-dimensional features. Users can trigger the LASSO operation, as shown in Fig. 3c, to select multiple points, and upon selection, other views trigger re-rendering simultaneously. Figure 3b shows that when the user hovers over a single point, the tooltip displays the name of the current regional industry, while the hover box in Fig. 3a shows its label name.

#### Geographical view

Depending on the design objective, we divided the geographical view into two sub-views: the glyph map view and the density map view. Users can switch between these two sub-views using a specific button on the control panel.

##### Glyph map view

**Fig. 4:**
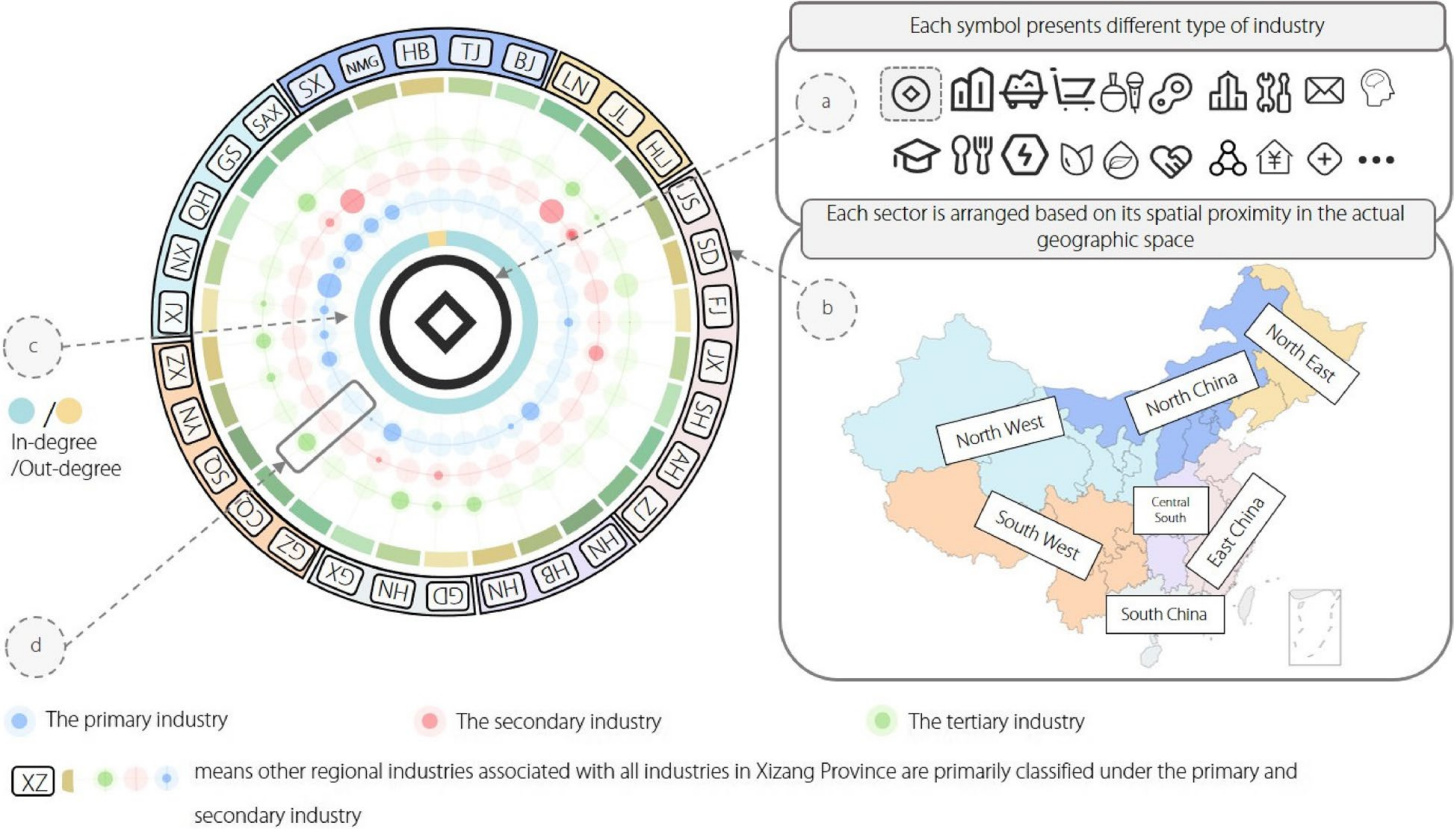
The designed metaphorical symbol is composed of four parts: **a** the icon presents the type of industry, **c** the donut chart displays the out-degree and in-degree of the current node, **d** three hollow circles present the number of connections among the current regional industry and industries in another region marked in (**b**)

Proper icons can not only quickly convey visual information to users compared to text but also effectively reduce visual overlap with other elements [26]. We propose a novel metaphorical symbol, as illustrated in Fig. 4, to aggregate information on the relationships between regional industries from multiple perspectives (R2). It had an overall shape similar to that of a wheel and consisted of four parts. The wheel-like shape was chosen considering the metaphorical symbol that we designed to represent the connections between regional industries. Simultaneously, we aimed to minimize the mutual overlap of network nodes on the map. In Fig. 4a, we utilize 20 icons [27] with sufficient distinctions to distinguish various industries. Figure 4c encodes the current node’s in-degree and out-degree in the RIN with blue and yellow, respectively. By comparing the size relationship between the node’s out-degree (  ) and in-degree (  ), users can intuitively understand the approximate position of the current regional industry in the whole RIN. Figure 4d includes a series of radial axes, and one radial axis consists of three fixed hollow circles, which represent the primary industry, secondary industry, and tertiary industry from inside to outside. The higher the opacity of the circle, the greater the number of connections between the current regional industry and a certain type of industry in another region. The spaced circles in Fig. 4b indicate the different regions. These regions were categorized into seven classes based on their geographical distribution and administrative planning. The relative positions of these classes determine the specific layouts of different modules on the circles.

Based on the calculated geographic locations, the symbol sizes were adjusted, and they were placed in the corresponding regions on the map. We use directed solid links on the map to represent the topology of the RIN because one of the main tasks of the system design is to visualize the connectivity in the RIN (R1). During the implementation process, we found that the number of edges in the RIN was excessively large. Directly displaying all edges in the view leads to severe overcrowding and overlap, resulting in visual clutter. Although there are methods to enhance the readability of the visual design [28], such as applying sampling methods [29] to the graph structures to retain only a few important structures, considering the task applicability (R2), we divide the edges in the RIN into “strong connections” and “weak connections” according to their weights. “Strong connections” refer to closer relationships between regional industries, such as the electronic device manufacturing industry in Sichuan Province and the battery manufacturing industry in Sichuan Province. “Weak connections” refer to relatively weaker relationships between regional industries, such as the electronic device manufacturing industry in Sichuan Province and the glass product manufacturing industry in Fujian Province. In the system backend, “strong connections” and “weak connections” were filtered based on whether the edge weight (in the range of 0–1) exceeds the threshold (k = 0.8). In the system frontend, users can control the display of strong connections in the glyph map view using the “Only Strong Connections” button in the control panel. Choosing to display only strong connections decreases the number of visible connections on the map, retaining the most important links while reducing visual clutter.

**Fig. 5:**
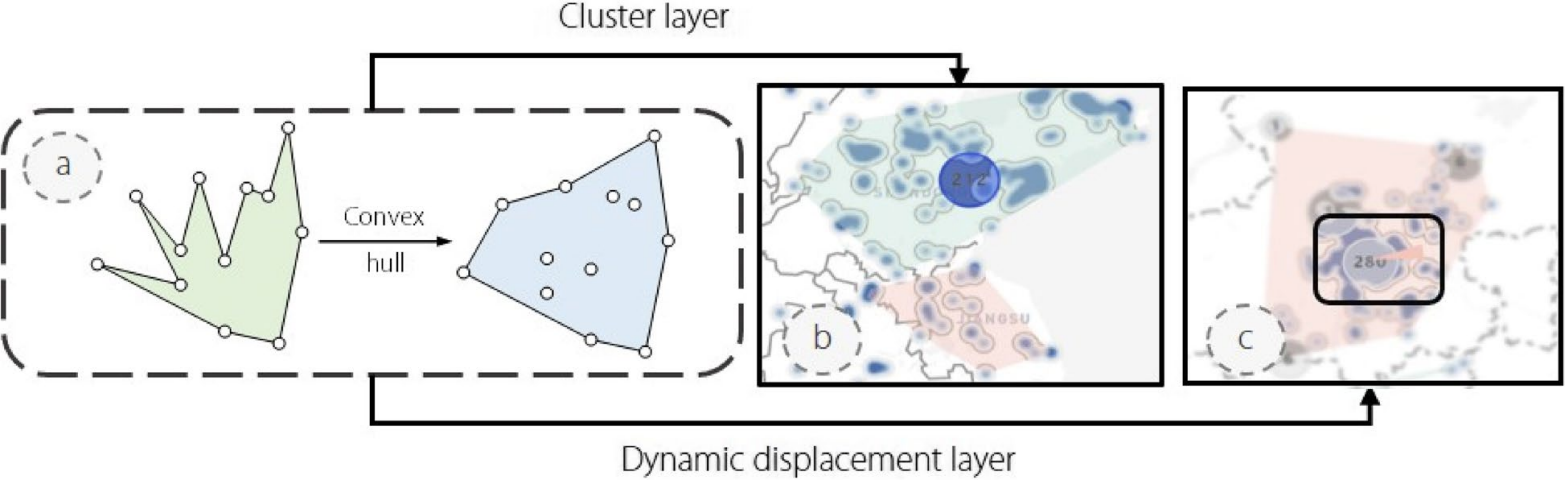
The density map view contains two layers. The cluster layer (**b**) shows the cluster of each regional industry through context hull algorithm (**a**). The dynamic displacement layer (**c**) presents regional industries’ movement direction at the next time point

##### Density map view

Compared to the glyph map view, which emphasizes the distribution of regional industry centroids and the correlation between regional industries, the density map view focuses on depicting the overall distribution of individual regional industries on the map and the direction of change in location at the next time point (R4). As illustrated in Fig. 5, the smallest draw unit of the heat map in the density map view is the company. We use the convex hull algorithm [30], as shown in Fig. 5a, to circle companies belonging to the same regional industry and display different clusters in different colors. When the user moves the mouse over the hover circle (Fig. 5b), the convex hull algorithm is automatically executed, and the result is plotted at the interface. The convex hull algorithm enables using the smallest polygons to wrap all elements in the cluster and reduce convexity defects. In addition, we drew the dynamic displacement layer as shown in Fig. 5c. We calculated the coordinates of the geographic center of each cluster year by year and used arrows to indicate the current cluster’s direction of position movement at the next time point. The color of the arrow is consistent with that of the cluster, and the tail and head of the arrow identify the coordinates of the geographic center of the cluster at the current and subsequent moments, respectively. In this innovative way, users can easily observe changes in the geographical distribution of different regional industries over time without switching between views belonging to different time points.

#### Timeline view

**Fig. 6:**
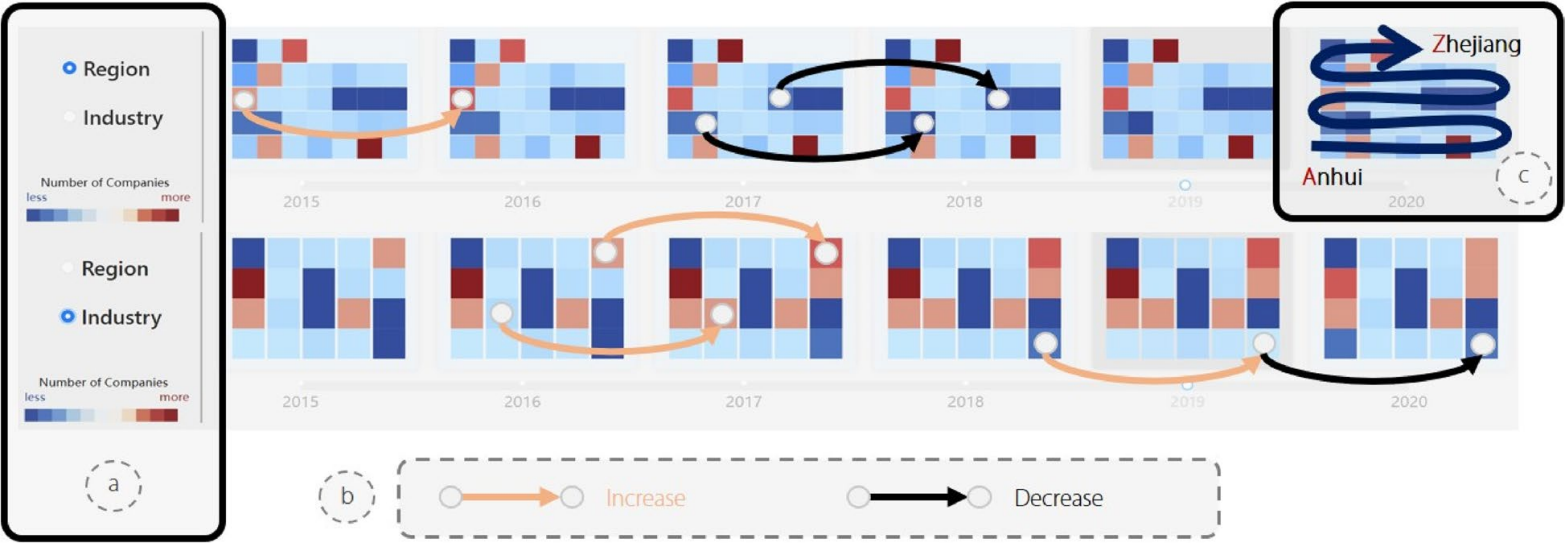
The timeline view combines time sliders and calendar heatmaps to display the number of companies over time. Users can switch blocks’ semantics in (**a**). Blocks are organized alphabetically according to their corresponding label names (**c**). We use gold and black lines (**b**) to respectively denote modules with significant changes

In addition to providing users with the ability to select specific time points, it is advantageous to allow them to effortlessly observe spatial and temporal variations of an entity’s scale across different regions or industries. To accomplish this, we integrated a calendar heatmap with a customized timeline. A series of blocks were organized alphabetically according to their corresponding label names at each time point. The varying colors of the blocks indicate the number of companies in their respective regions or industries. Users can autonomously switch the blocks’ semantics by clicking a button on the left. Whether clicking on a block or timeline, both trigger an automatic re-rendering of other views. In Fig. 6, we use the gold and black lines to highlight the blocks in which the number of companies exhibits significant increases and decreases between adjacent time points. By enhancing the traditional timeline, users can explore several companies more intuitively over time.

#### Statistical view

Considering the user’s requirement to observe the high-dimensional attributes of each regional industry entity and recognizing that the projection methods used in the semantic map view lack explanatory power in this regard, parallel coordinates were selected to present the trend of important economic indicators of selected regional industries over time. Based on expert recommendations, six indicators were summarized from five dimensions: economic development capability (gdp and per), credit performance (kmv), attractiveness to talent (attractiveness), sustainability (sustainable), and high-tech development capability (high-tech) to provide a comprehensive analysis of the regional industry. As shown in Fig. 2e, different coordinates indicate different indicators and different color curves indicate different time points. Thus, users can explore in detail the economic development preference of each regional industry and the changes in its indicators per dimension over time.

## Results

To validate our visual analytic approaches, we conducted two case studies and interviewed experts on the user experience.

### Case 1: semantic-oriented analysis of regional industries

In the first case study, utilizing V4RIN to explore regional industries with similar economic characteristics was illustrated.

**Fig. 7:**
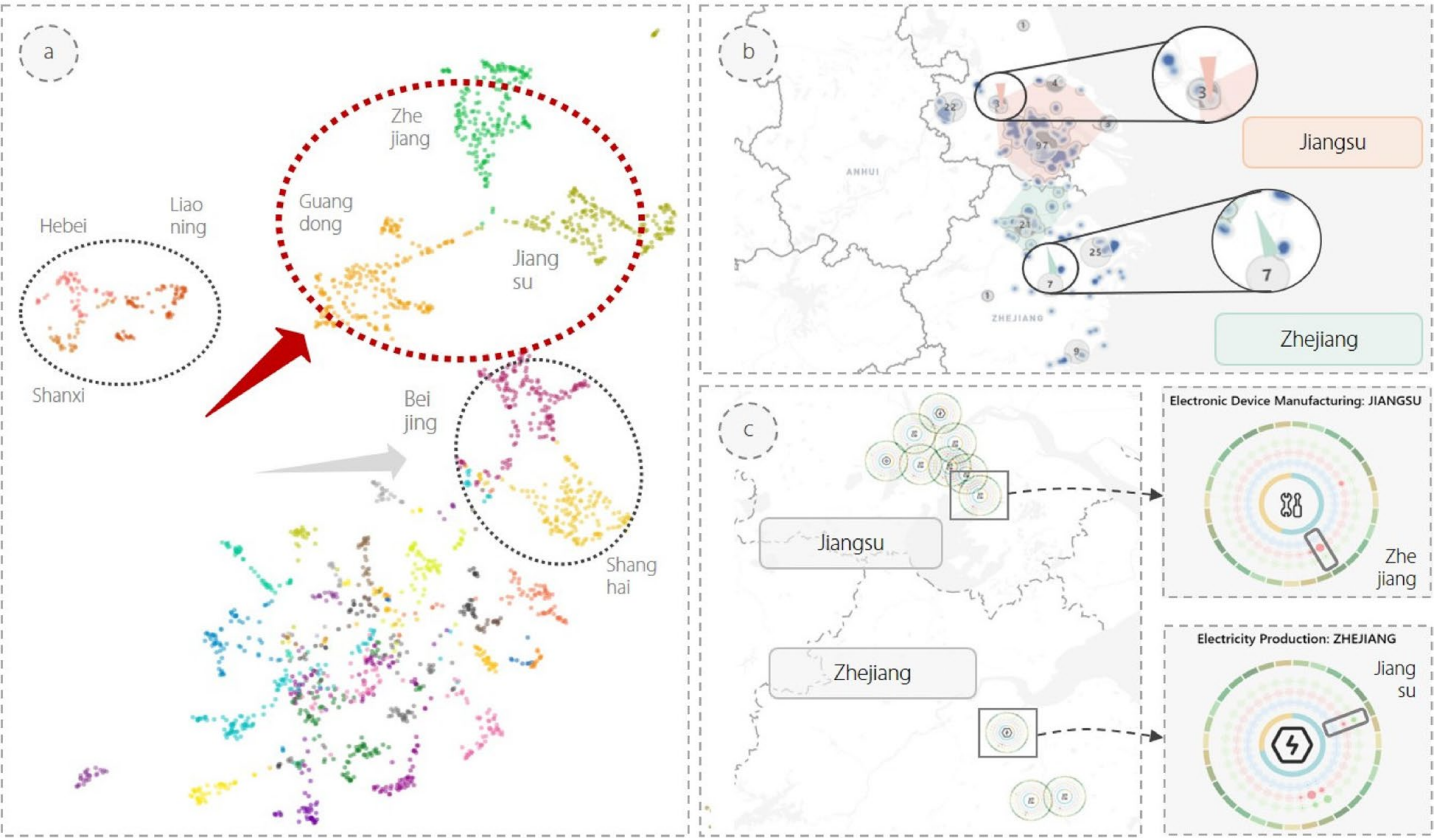
Several regions are clustered together in the semantic projection view (**a**). After selecting regional industries in Jiangsu and Zhejiang Provinces, the arrows (**b**) show that they tend to be closer to each other in the future. The glyph map view (**c**) displays their detailed metaphorical symbols in the map

First, the semantic projection view depicts an evident aggregation effect of points on the scatterplot when a region is used as the label. For example, as shown in Fig. 7a, the dimensional reduction results for regional industries in Guangdong, Zhejiang, and Jiangsu Provinces are significantly closer together. There is also a stronger correlation between regional industries located in Beijing and Shanghai. After selecting some regional industries located in Zhejiang and Jiangsu Provinces with similar semantics using the LASSO operation, Fig. 7b shows the distribution of all listed companies in the selected regional industries on the map. The filtered regional industries located in Jiangsu and Zhejiang Provinces have more neighboring geographical distributions. By intensively viewing the information of listed companies belonging to these regional industries through the statistic view, it is observed that there is indeed a strong semantic association between these companies, such as Trina Solar in Jiangsu Province, engaged in transmission and distribution, and control equipment manufacturing, and Zhejiang Province’s zhehai deman, engaged in automotive parts and accessories manufacturing. Additionally, the pink and light green arrows in Fig. 7b identify the geographical trends of these regional industries, respectively. We found that they tend to be closer to each other in the future. This is in line with the development plan of each industry for Jiangsu and Zhejiang Provinces in the “Outline of the Yangtze River Delta Regional Integrated Development Plan” released by the State Council in December 2019. This indicates that our system can effectively capture the response of each regional industry to a policy regime.

The metaphorical symbols characterizing these regional industries were then drawn in the glyph map view based on the pre-calculated geographic coordinates, as shown in Fig. 7c. It was observed that the electricity production industry in Zhejiang Province has a relatively dense association with Jiangsu Province, whereas the electronic device manufacturing industry in Jiangsu Province has a relatively dense association with Zhejiang Province. Using this approach, experts can easily observe which regional industries share close semantic connections with the target regional industry and possess higher investment potential. In summary, this case study demonstrates how the proposed system can facilitate the exploration of the inherent economic characteristics of regional industries, discovering the distribution and interrelationships among regional industries with similar economic characteristics on the map.

**Fig. 8:**
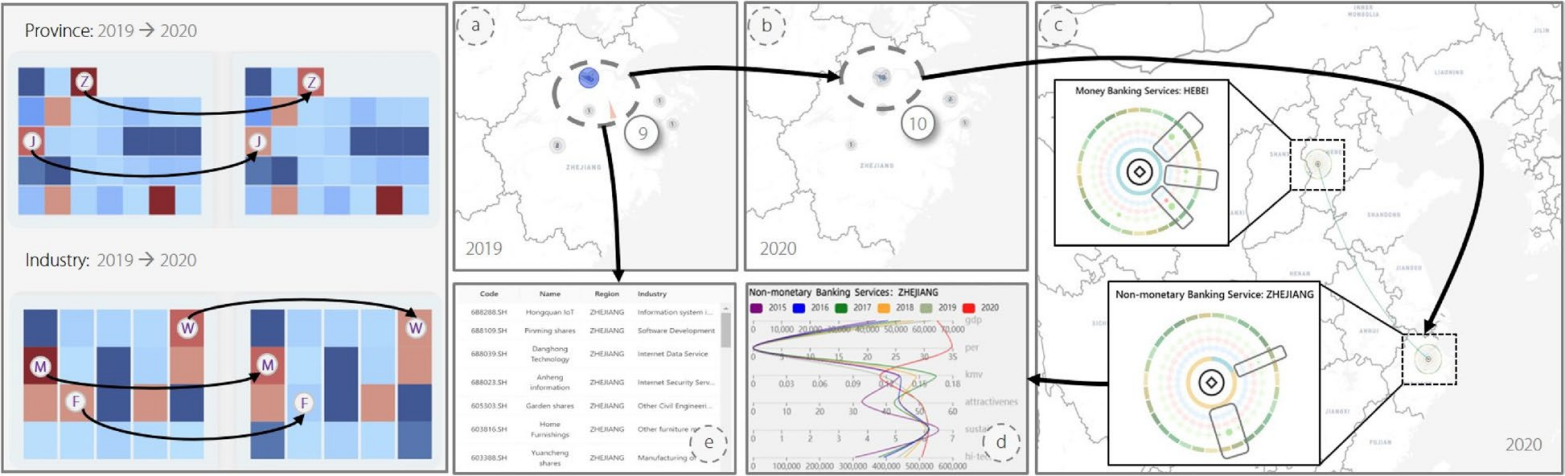
The left side depicts the changes in the number of companies in each province and industry from 2019 to 2020. It is noticeable that there is a significant decrease in the number of companies in Jiangsu (J) and Zhejiang (Z) provinces after 2019. The number of companies engaged in the financial (F), manufacturing (M), and wholesale (W) sectors also shrank to varying degrees. From (**a**) to (**b**), more financial companies located in Zhejiang Province are clustered in Hangzhou. The financial industries in Zhejiang Province have stronger connections with the financial industries in Hebei Province (**c**). Moreover, the statistical view presents some important indicators of selected regional industries (**d**) and the basic information of listed companies belonging to them

### Case 2: spatio-temporal analysis of RIN

As COVID-19 has had a significant impact on the global economy, leading to a sharp decline in economic activities and disruption of global supply chains, domain experts are curious about the trends in economic characteristics and geographical distributions of regional industries around 2019, and whether the connections between these industries had a significant impact on their development. Therefore, in the second case study, the dynamic evolution of the regional industry’s own characteristics and the connections between them around 2019 were analyzed.

On the left of Fig. 8, there is a significant decrease in the number of listed companies in Jiangsu and Zhejiang Provinces in 2020 compared with 2019. Meanwhile, the number of companies engaged in the financial, manufacturing, and wholesale sectors shrank to varying degrees. Therefore, the financial industries in Zhejiang Province were analyzed. As shown in Fig. 8a and b, the financial industry in Zhejiang Province is geographically distributed with a provincial capital concentration. Compared with 2019, more financial companies located in Zhejiang Province were clustered in Hangzhou in 2020, indicating that companies tended to look for development opportunities in the provincial capital city with more resources under the influence of the epidemic. When selecting the non-monetary banking service of Zhejiang Province on the map, Fig. 8c illustrates that this regional industry has a greater out degree in RIN, which indicates that it is closer to the upstream of the network and is more likely to be a product provider. It has a more intensive association with the tertiary industry in Hunan, Jiangsu, and Guangdong Provinces. This is in line with the actual geographical distribution of the non-monetary banking service industry and the requirements of the “Business Development Plan for the 13th Five-Year Plan Period”. When the other regional industries associated with it need to be seen, the “Show Relationships” switch is turned on in the Control Panel and the map is automatically redrawn. The economic connections between regional industries are plotted in a new layer using these curves. It can be seen that it has a more significant relationship with the industry in Hebei Province by selecting Non-Monetary Banking Services in Zhejiang Province in Fig. 8c. Simultaneously, as shown in Fig. 8e, the statistical view presents the basic information on the listed companies belonging to the financial industry in Zhejiang Province, helping experts quickly obtain information about leading companies in the regional industry. In addition, the statistical view to further examine the trends of some important economic indicators of the financial industry in Zhejiang Province over the last six years can be explored. As shown in Fig. 8d, for the non-monetary banking services industry in Zhejiang Province, its kmv default probability decreased significantly in 2020 compared to the previous five years, and its gdp, attractiveness, and hi-tech values increased somewhat, which indicates that it has a good development trend and investors can pay more attention to it.

### Expert review

As the visual analytic system was designed for professional financial analysts, a set of expert interviews was conducted to evaluate the usability of the system. All interviewees–two specialists E4 and E5 from one financial company and four researchers E6-E9 in the field of fintech–had in-depth knowledge of regional economics or industry economics. A detailed demonstration of each view in the system and its functionality was provided to ensure the experts’ understanding of the system’s capabilities. Interviewees were then allowed to freely explore and interact with the system, after which individual interviews were conducted to gather their queries and feedback on how to effectively utilize the system.

The interview questions were divided into three perspectives. (1) Importance. Is the core issue of the work important? Does it make sense to use visual analytic tools to address complex financial problems? (2) Effectiveness. Does the exploratory function of this system rely on high domain knowledge? Is it easy to observe the RINs using a graph-based geovisualization approach? (3) Usability. Is the functional design of each sub-interface reasonable and understandable? Is the set of interactions in the system user-friendly?

#### Importance

E4 stated that in practice, visual analytical tools for RINs could effectively assist in policy recommendations, regional management, and industrial upgrades. E7 commented, “it is theoretically and practically useful to highlight the connections between them via graph-based geovisualization.” Regarding the use of visual analytic systems to deal with complex financial problems, E6 said, “complex financial problems often involve complex data that is challenging even for professionals to handle. Visual analytics can enhance the comprehensibility of the data.”

#### Effectiveness

E5 noted that the views visually reflected changes in the data and conveyed important information to the users. Regarding the visual presentation of the RIN in the system, E8 stated that the density of the links allowed users to visually explore the economic connections between different regions and industries. For example, he discovered a relatively strong economic link between the technology service industry in Shanghai and the financial service industry in Xinjiang Uygur Autonomous Region. This potential association is often overlooked in the daily analyses of experts. We appreciate the experts’ high appreciation of our work and their expectations for the future. We also expressed our commitment to continue enhancing this work and applying it to a broader range of financial tasks in the future.

#### Usability

E8 stated that the designed metaphorical symbol is not only beautiful and understandable, but also integrates diverse information in a hierarchical structure so that “without complex interactions, I can visually understand how a region’s industries are related to other regions or industries on one map at the same time.” Regarding the system’s interaction, all experts believed that the range of interactions provided in the system was easy to operate and human-machine friendly. In addition, E9 pointed out the blurring of the magnification of the metaphorical symbols on the map and the overlapping problem. After explaining the technical limitations used and showing the number of regional industries without adequate filtering, they understood the difficulty of balancing the accuracy of the information with the visual impact. We will continue to improve our system in the future based on the valuable feedback provided by the experts.

## Discussion

The domain experts were closely collaborated with to analyze the RIN, which is challenging to describe directly using statistical data. Learning from experts’ research experience, a series of domain tasks for regional industry analysis was summarized. The proposed pipeline and visualizations are specifically designed to address these tasks, aiding experts in analyzing various aspects of regional industry entities’ economic status, spatio-temporal evolution, the intensity of their economic connections, etc. The proposed visual analytic methods are applicable to RINs, addressing shortcomings in previous financial visualization tools in handling domain-specific analysis tasks, such as the difficulty of concisely identifying the connectivity between regional industries and the inability to synthesize the geographical distribution of regional industries and their evolutionary trends over time within the same view. Although case studies and expert interviews have demonstrated the effectiveness of the proposed approach in addressing the tasks faced by domain experts when analyzing RINs for relationships among other financial entities with heterogeneous characteristics, such as stock networks [31], the core data processing algorithms and related visual designs can be adaptively applied because of their inherent structural similarities with RINs. In addition, the proposed visual solution can be extended to other financial analysis tasks. For instance, in a portfolio analysis, investors and asset managers can consider different financial institutions as nodes and treat the interconnections and dependencies between these institutions as edges. By inputting well-constructed network data into the system, the interface can assist users in interactively exploring institutional distribution and connection patterns from various perspectives, thereby supporting risk assessment and decision-making. Furthermore, through ongoing discussions with experts, some drawbacks of the current visual designs were identified. Therefore, targeted solutions that can be implemented in the future were proposed.

### Enhance visual designs to provide greater flexibility and customized options

Although the proposed visual designs primarily serve the domain-specific tasks proposed in Requirement analysis subsection, fixed data presentation formats may limit the usability and scalability of visual analytic tools because of users’ diverse perspectives and goals in exploring RINs. For example, the parallel coordinates view only provides the temporal variations of five fixed economic indicators for individual regional industry entities. Some experts suggested incorporating more indicators and supporting users in selecting economic indicators independently for comparison. Therefore, in the future, current visual designs will be enhanced by offering users more choices and customization options for data representation.

### Expand the current geographical visualization approach to higher dimensions

A geographic view based on 2D maps was designed to present the geographical distribution of regional industries. However, such visual representation forms, when combined with layers of graphical symbols, line graphs, or dynamic displacement layers, can easily lead to information overcrowding, hindering the experts’ ability to interact conveniently. Inspired by Sabrina [3], the future plan involves adding 3D effects and local zooming operations to enhance the expressiveness of the data on a flat surface.

### Improve the layout of nodes and edges on the map

Although current methods improve graph-based geovisualization by connection strength and metaphorical symbols, experts still find it difficult to analyze directly on a map because the node distribution is too dense in some regions. In the future, improved edge aggregation or binding algorithms to optimize the visual perception will be applied.

### Optimize interface rendering speed and interaction response time

After conducting timing statistics, experts, on average, spend 3.7 min proficiently completing a moderately complex RIN analysis task, such as identifying regional industries with similar economic characteristics and observing their spatial distribution and temporal changes on the map. Some experts have indicated that the performance bottleneck mainly lies in the delay of the overall interface rendering after manipulating the visual representation of subviews, which hinders their analytical workflow. Therefore, the visual engine will be optimized by leveraging system development knowledge to accelerate the interaction response time of the interface.

## Conclusions

In this study, in collaboration with domain experts, challenges were identified and domain tasks in a RIN analysis were summarized. An interactive visualization analysis system, V4RIN, was designed and implemented, integrating data-processing methods and several visual analysis views. These visual designs can assist experts in exploring the relationships among regional industries and their growth potential, enabling informed decisions on investments, workforce planning, and so on. The proposed system is a new visual analytical approach for RINs using domain knowledge. Two case studies and a set of expert interviews demonstrated the effectiveness of decision-making and communication derived with V4RIN.

## Data Availability

The datasets generated and/or analysed during the current study are not publicly available due to personal privacy but are available from the corresponding author on reasonable request.

## Abbreviations

RIN: Regional industry network
V4RIN: An interactive visualization analysis system for regional industry network
JSON: JavaScript object notation
GNN: Graph neural network
